# Comparative Effects of Inorganic Additives on the Filtration Performance of PVDF-Based Electrospun Air Filters

**DOI:** 10.3390/nano16161023

**Published:** 2026-08-18

**Authors:** Chanwoo Park, Dohyoung Kang, Hobin Jee, Yebin Hong, Changhyuk Kim, Sukbyung Chae, Jungmin Lee, Soonchul Kwon, Ji Yong Park, Numan Yanar, Euntae Yang

**Affiliations:** 1 Department of Marine Environmental Engineering, Gyeongsang National University, Tongyoung 53064, Republic of Korea; pcw0112@gnu.ac.kr (C.P.); ybh03@gnu.ac.kr (Y.H.); 2 Department of Civil and Environmental Engineering, Pusan National University, Busan 46241, Republic of Korea; changhyuk.kim@pusan.ac.kr (C.K.); sckwon@pusan.ac.kr (S.K.); 3 Institute for Environment and Energy, Pusan National University, Busan 46241, Republic of Korea; 4 Department of Mechanical Engineering, Korea University of Technology and Education, Cheonan 31253, Republic of Korea; schae@koreatech.ac.kr; 5 Land & Housing Institute, Korea Land & Housing Corporation, Daejeon 305713, Republic of Korea; 6 Nexmore Systems Co., Ltd., Ansan 15426, Republic of Korea; 7 R&D Center, Naieel Technology, Daejeon 34026, Republic of Korea

**Keywords:** electret air filter, electrospun nanofiber, band gap, dielectric constant, inorganic additives

## Abstract

Electrospun poly(vinylidene fluoride) (PVDF) nanofiber filters are attractive for particulate air filtration because they combine high filtration efficiency with low airflow resistance. Here, three inorganic additives, namely aluminum chloride (AlCl_3_), potassium nitrate (KNO_3_), and silicon nitride (Si_3_N_4_), were investigated using formulation-specific electrospinning conditions selected to achieve stable fiber formation. Although all filters exhibited high initial filtration efficiencies above 96%, clear differences were observed in fiber morphology, pressure drop, electrostatic potential decay, and long-term filtration stability. Among the tested samples, the Si_3_N_4_-containing filter showed the best long-term performance, retaining approximately 94% filtration efficiency after 30 days, whereas neat PVDF decreased to about 85%. These results suggest that differences in long-term filtration stability are closely associated with charge-retention behavior, and that the incorporation of inorganic additives can influence the durability of PVDF-based electrospun nanofiber air filters.

## 1. Introduction

Currently, fibrous air filters, including melt-blown and electrospun types, are one of the most effective approaches for particulate matter removal owing to their high filtration efficiency, portability, mobility, and cost-effectiveness [1,2,3,4]. These filters capture particles through a combination of mechanical mechanisms, such as interception, impaction, and diffusion, as well as electrostatic attraction [4]. Accordingly, the performance of fibrous air filters can be improved by modifying either of these filtration mechanisms. Although enhancing mechanical filtration is the simplest approach to improving filtration efficiency, it often restricts airflow, resulting in increased pressure drop and reduced overall filter performance [5,6]. Therefore, strategies that improve filtration efficiency while minimizing this trade-off are highly desirable in air filter development [7].

One of the most widely adopted strategies to address this challenge is the incorporation of additives into polymer matrices. Numerous studies have reported that inorganic additives can be introduced into polymer nanofiber matrices to improve the filtration performance and physicochemical properties [8,9]. For example, Ke et al. [10] prepared electrospun poly(lactic acid) (PLA)/ zinc-doped titanium dioxide (Zn-doped TiO_2_) nanofibrous filters, in which Zn doping reduced the TiO_2_ bandgap from 3.2 to 3.0 eV, resulting in enhanced charge separation and dielectric properties. Consequently, the surface potential of the PLA nanofibers increased to ~5.7 kV, while the fiber diameter decreased from 581 nm to 264 nm, leading to enhanced electrostatic adsorption and mechanical interception and achieving a filtration efficiency of 98.7% for particulate matter with a diameter of 0.3 μm (PM0.3). Wang et al. [11]. incorporated polytetrafluoroethylene (PTFE) nanoparticles (0.05 wt.%) into electrospun poly(vinylidene fluoride) (PVDF) nanofibers, which deepened the energy levels of charge traps and increased dipole and interfacial polarization charges, thereby enhancing the surface potential from 0.42 to 3.63 kV. As a result, the resulting electret nanofibrous membrane achieved a high PM2.5 filtration efficiency of 99.97% with a low-pressure drop of 57 Pa. Wang et al. [8]. developed a biodegradable fiber membrane (SF/Wool) by introducing silk fibroin (SF) onto the surface of wool fibers, which enhanced interfacial polarization and increased the relative dielectric constant from 4.4 to 8.4. As a result, the SF/Wool membrane achieved a PM0.3 filtration efficiency of 99.69% with an ultralow pressure drop of 8 Pa, while exhibiting excellent long-term stability with less than 0.5% efficiency loss over 30 days. Ding et al. [9]. uniformly dispersed γ-glycidoxypropyl trimethoxy silane-modified silicon dioxide nanoparticles (GPS@SiO_2_) within electrospun PVDF nanofibers, thereby generating abundant interfacial charges and realizing enhanced electret properties. As a result, the resulting membrane exhibited a high surface potential of 12.4 kV along with an excellent filtration performance with a quality factor (QF) of 0.14 Pa^−1^.

Among electrospun polymers, PVDF has been widely used for air filtration because of its high molecular polarity, chemical and thermal stability, and inherent electret properties [12]. Previous studies have improved PVDF filter performance through both additive modification and structural design. Negative-ion powder and magnetite (Fe_3_O_4_) have been introduced to enhance electrostatic or magnetic particle capture [13,14], while surfactants such as sodium dodecylbenzene sulfonate (SDBS) and sodium dodecyl sulfate (SDS) have been used to reduce fiber diameter and form ultrafine fiber or nanonet structures [15,16]. Multilayer PVDF nanofiber filters have also been developed to improve nano-aerosol filtration, particle-loading behavior, and electrostatic charge retention [17,18,19], while three-dimensional curly fibrous structures have been introduced to increase porosity and reduce airflow resistance [20]. More recent studies have further evaluated the stability of PVDF-based filters under chemical, thermal, humid, and prolonged outdoor exposure conditions [15,21]. However, previous studies have generally focused on optimizing a single additive or filter structure, and systematic comparisons of different additive types within the same PVDF matrix remain limited. In particular, ionic salt additives may mainly affect solution conductivity and fiber formation, whereas dielectric ceramic additives may contribute to interfacial polarization and charge retention. These effects have rarely been directly compared under consistently controlled fabrication, storage, and filtration-test procedures.

Based on this research gap, aluminum chloride (AlCl_3_), potassium nitrate (KNO_3_), and silicon nitride (Si_3_N_4_) were selected as inorganic additives with distinct properties and expected interaction mechanisms. AlCl_3_ is a multivalent ionic salt that can modify the conductivity of the spinning solution and its interactions with polymer chains. The addition of aluminum chloride hexahydrate (AlCl_3_·6H_2_O) to a poly(vinylidene fluoride-co-hexafluoropropylene) [P(VDF-HFP)] electrospinning solution has been reported to alter the fiber morphology, crystalline-phase composition, and dielectric properties; however, air-filtration performance was not evaluated in that study [22]. KNO_3_ is also an ionic salt that can affect solution conductivity and electrohydrodynamic jet behavior. PVDF/KNO_3_ composite sub-microfibers have previously been prepared through solution blow spinning, but the resulting material was developed as a controlled-release matrix for fertilizer rather than for air filtration [23]. Unlike these salt-type additives, Si_3_N_4_ is a dielectric ceramic particulate additive that may contribute to polarization and charge stabilization at polymer–particle interfaces. Jiang et al. increased the stable surface potential of poly(vinyl butyral) (PVB) nanofibers from 1610 to 2010 V by adding 1 wt.% Si_3_N_4_. After the further introduction of fluorinated polyurethane (FPU), the resulting PVB/Si_3_N_4_–FPU membrane exhibited a filtration efficiency of 99.95% with a pressure drop of 55 Pa [24]. Liu et al. also prepared an electrospun polyurethane (PU)/Si_3_N_4_ membrane and demonstrated the contribution of Si_3_N_4_ to the electret and air-filtration properties. The resulting filter exhibited a filtration efficiency of 79.36% with a pressure drop of 25 Pa [25]. However, these studies used PVB or PU as the polymer matrix, and the effects of Si_3_N_4_ on the formation and long-term filtration performance of PVDF nanofiber filters have not been directly investigated.

Taken together, AlCl_3_ and KNO_3_ have been incorporated into PVDF or PVDF-copolymer-based fibrous materials, but their air-filtration performance has not been evaluated. Si_3_N_4_ has been applied to PVB- and PU-based electret filters, but no previous study applying Si_3_N_4_ to a PVDF-based air filter was identified. Moreover, the effects of AlCl_3_, KNO_3_, and Si_3_N_4_ have not been directly compared within the same PVDF electrospinning system. Therefore, PVDF was used as a common polymer matrix in this study to systematically compare the effects of the ionic salt additives AlCl_3_ and KNO_3_ with those of the dielectric ceramic particulate additive Si_3_N_4_ on electrospinning and air-filtration performance. Solution conductivity and viscosity were measured to interpret the differences in fiber morphology associated with the additives. Chemical composition, crystalline structure, surface properties, initial filtration performance, and performance retention over 30 days were subsequently evaluated using consistently controlled fabrication, storage, and filtration-test procedures.

## 2. Materials and Methods

### 2.1. Preparation of PVDF Solutions for Electrospinning with Various Inorganic Additives

**Figure 1:**
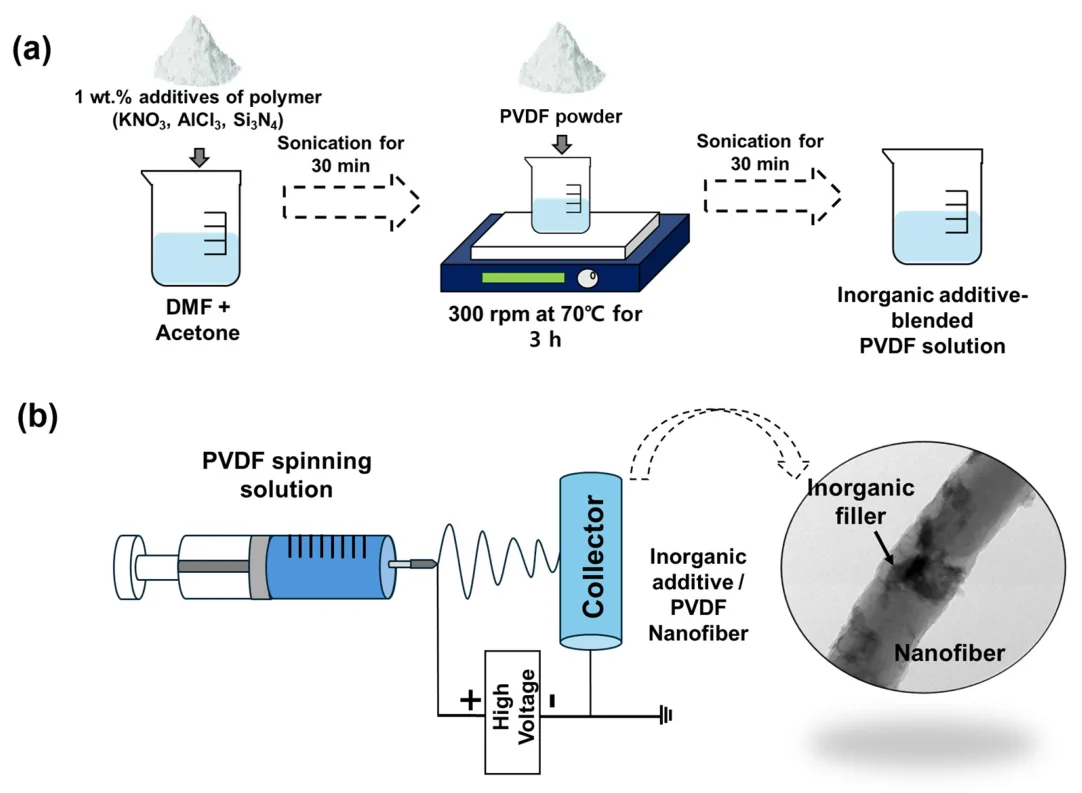
Schematic illustration of (**a**) the preparation of inorganic filler-–blended PVDF solution solutions and (**b**) the electrospinning process using the prepared dope solution and resulting distribution of inorganic additives within a PVDF nanofiber.

PVDF (Sigma-Aldrich, St. Louis, MO, USA) was used as the polymer matrix. AlCl_3_ (anhydrous, reagent grade, 98%, Sigma-Aldrich, USA), KNO_3_ (ACS reagent, ≥99.0%, Sigma-Aldrich, USA), and Si_3_N_4_ (nanopowder, <50 nm particle size, ≥98.5% trace metals basis, Sigma-Aldrich, USA) were used as inorganic additives. The supplier did not specify the particle sizes of AlCl_3_ and KNO_3_. N,N-Dimethylformamide (DMF) and acetone were purchased from Daejung Chemicals & Metals Co., Ltd. (Siheung, Republic of Korea). After selecting the optimal polymer concentration, spinning solutions were prepared by incorporating different inorganic additives at 1 wt.% relative to the polymer in order to comparatively evaluate their effects on fiber formation, filtration performance, and storage-time-dependent performance retention (Figure 1a). Table 1 summarizes characteristics of the selected inorganic additives.

All polymer solutions were sonicated at 40 kHz for 30 min using an ultrasonic processor (Powersonic 510, Hwashin, Seoul, Republic of Korea) and subsequently stirred at 70 °C and 300 rpm for 3 h. The nanofiber air filters were named according to the incorporated materials. Neat PVDF, AlCl_3_-blended PVDF, KNO_3_-blended PVDF, and Si_3_N_4_-blended PVDF nanofiber air filters were denoted as N-PVDF, A-PVDF, K-PVDF, and S-PVDF, respectively. The detailed fabrication parameters are provided in Table 2.

The conductivity of each spinning solution was measured using a CON 2700 conductivity meter equipped with a CONSEN9501D probe (Eutech Instruments, Singapore). Viscosity was measured using a DV-II Pro viscometer (Brookfield Engineering Laboratories, Inc., Middleboro, MA, USA) at a rotational speed of 10 rpm. The measured solution properties and detailed electrospinning conditions are summarized in Table 3.

### 2.2. Fabrication of Nanofiber Air Filter Using Electrospinning

Nanofiber air filters were fabricated using a customized electrospinning system (Nano NC, Seoul, Republic of Korea). Polypropylene (PP) and polyethylene terephthalate (PET) nonwoven fabrics were initially examined as supporting layers, and PP was selected because of its better nanofiber attachment and mechanical stability. For the comparative experiments, the PVDF concentration, additive loading, solution volume, flow rate, nozzle gauge, tip-to-collector distance, deposition time, and collector area were maintained consistently for all formulations. Electrospinning was performed using a 24G metal nozzle at a flow rate of 5 mL h^−1^, a solution volume of 1 mL, a deposition time of 12 min, and a tip-to-collector distance of 15 cm.

The applied voltages for N-PVDF, A-PVDF, K-PVDF, and S-PVDF were 21, 21, 18, and 21.7 kV, respectively. Because the additives changed the conductivity and viscosity of the spinning solutions, the applied voltage was adjusted for each formulation to obtain a stable Taylor cone and continuous fiber formation. Therefore, formulation-specific stable electrospinning voltages were used rather than identical applied voltage. The solution volume, flow rate, deposition time, and collector area were maintained consistently for all formulations to control the nominal amount of spinning solution supplied and the deposition conditions. However, the actual deposited nanofiber mass was not measured separately; therefore, the deposited mass per unit area, bulk porosity, and packing density were not quantitatively determined. The detailed fabrication conditions and measured solution properties are summarized in Table 2 and Table 3, respectively.

### 2.3. Characterization of Air Filters

The surface and cross-sectional morphologies of the nanofiber air filters were examined using field-emission scanning electron microscopy (FE-SEM; Apreo 2S, Thermo Fisher Scientific, Brno, Czech Republic). Fiber diameters were measured using ImageJ software (version 1.54g, National Institutes of Health, Bethesda, MD, USA) by analyzing at least 50 individual fibers for each filter. Cross-sectional SEM images were used to examine the deposited nanofiber-layer structure and estimate the representative layer thickness using high precision calipers. The layer thickness was additionally measured using a high-precision digital micrometer (293-240-30, Mitutoyo, Kawasaki, Japan). The apparent surface pore size and projected pore-area fraction were determined from top-view SEM images using ImageJ. Images obtained at the same magnification were calibrated using their scale bars and converted into binary images using the same image-processing procedure. The projected area of each enclosed pore opening was measured, while pores intersecting the image boundaries were excluded from the pore-size analysis. The equivalent pore diameter (*d_eq_*) was calculated using the following equation.

$$d_{eq}=2\sqrt{\frac{A}{\pi }}$$

where A is the two-dimensional projected pore area. The projected pore-area fraction was calculated as the ratio of the thresholded pore area to the total analyzed image area. Because this analysis was based on two-dimensional SEM images, the obtained values represent apparent surface pore openings and projected pore-area fractions rather than through-pore diameters or bulk porosity.

The chemical composition and distribution of the inorganic additives in the PVDF nanofibers were examined using energy-dispersive X-ray spectroscopy coupled with FE-SEM (SEM–EDX; Apreo 2S, Thermo Fisher Scientific, Brno, Czech Republic). Elemental mapping was conducted for the elements related to each additive, including Al and Cl for A-PVDF, K and N for K-PVDF, and Si and N for S-PVDF. The distribution of the inorganic additives within the nanofibers was further examined using transmission electron microscopy (TEM; Tecnai F30 S-Twin, FEI, Hillsboro, OR, USA) operated at an accelerating voltage of 300 kV. Prior to TEM analysis, the nanofibers were dispersed in ethanol and deposited onto TEM grids. X-ray diffraction patterns of the PP substrate, neat PVDF, and additive-containing PVDF air filters were obtained using an X-ray diffractometer (XRD; Ultima IV, Rigaku Corporation, Akishima, Japan). The diffraction patterns were collected over a 2θ range of 5–100° with a step interval of 0.02° to examine the crystalline characteristics of PVDF and the incorporated inorganic additives.

The surface wettability of the nanofiber air filters was evaluated using a contact-angle goniometer (Phoenix 300, S.E.O, Suwon, Republic of Korea). The surface potential was measured using an electrostatic voltmeter (Model 542A, Trek, Lockport, NY, USA) at five different locations on 10 × 10 cm^2^ filter samples. The measurements were conducted immediately after fabrication and after 15 and 30 days of storage to evaluate the electrostatic charge-retention behavior of the filters. During the storage period, the filters were maintained in an isolated environment at room temperature and a relative humidity of 30–40%.

### 2.4. Filtration Performance Evaluation of Nanofiber Air Filters Using Automated Filter Tester

Filtration performance, including filter efficiency and pressure drop, was evaluated using an automated filter tester (TSI 8130, TSI Inc., Shoreview, MN, USA) at a flow rate of 32 L min^−1^ with polydisperse NaCl aerosols. Test aerosols were generated from a 2 wt.% NaCl solution and exhibited a mass median diameter of 260 nm, a count median diameter of 75 nm, and a geometric standard deviation (GSD) below 1.86. The TSI 8130 is a widely accepted platform for evaluating the particulate filtration efficiency and pressure drop of respirator filters and filter media. The use of this instrument also facilitates comparison with prior literature on electrospun nanofiber-based air filtration materials [4,26,27]. In addition, the NaCl aerosols with nanoscale particles provide a stringent condition close to the most penetrating particle size, ensuring reliable assessment of electrostatic filtration behavior.

The air filters were tested at a face velocity of 5.33 cm s^−1^ with a fixed test area of 100 cm^2^, while maintaining a constant flow rate of 32 L min^−1^. To investigate time-dependent changes in filtration performance associated with the incorporated inorganic additives, measurements were conducted immediately after fabrication (day 0) and after 15 and 30 days of storage under controlled conditions (relative humidity of 30–40% and room temperature) in an isolated environment.

Filtration efficiency for each filter was calculated as the average of three measurements taken at different locations on the filter. The reported values represent the mean and standard deviation obtained from five independently prepared samples for each filter.

To provide an integrated assessment of filtration performance, the *QF* was also calculated from the measured filtration efficiency (*θ*) and pressure drop (Δ*P*) using the following equation [10]:

$$QF=-\frac{ln(1-\theta )}{∆P}$$

**Table 1:** Characteristics of the inorganic additives used in this study.

Additive	Dielectric Constant (ε)	Band Gap (eV)	Additive Type	Likely Form in Spinning Dope	Reference
Aluminum chloride (AlCl_3_)	2.68	6.05	Salt-type inorganic additive	Dissolved or strongly solvent-interacting additive	[22,28]
Potassium nitrate (KNO_3_)	5.00	3.80	Salt-type inorganic additive	Salt-type additive in the spinning dope	[29,30]
Silicon nitride (Si_3_N_4_)	7.50	5.30	Ceramic inorganic additive	Dispersed particulate additive	[31,32]

**Table 2:** Electrospinning fabrication parameters for neat and inorganic filler-incorporated PVDF air filters.

Filter	PVDF Content (wt.%)	Inorganic Additive (1 wt.%)	Voltage (kV)	Nozzle Gauge	Flow Rate (mL h^−1^)	TCD ^(1)^ (cm)	Solution Volume (mL)	Deposition Time
N-PVDF	12–18	None	21.0	24G	5	15	1	12
A-PVDF	12	Aluminum chloride	21.0	24G	5	15	1	12
K-PVDF	12	Potassium nitrate	18.0	24G	5	15	1	12
S-PVDF	12	Silicon nitride	21.7	24G	5	15	1	12

^(1)^ Tip-to-collector distance (cm).

**Table 3:** Electrical conductivity and viscosity of the spinning solutions.

Sample	Conductivity (μS cm^−1^)	Viscosity (cP)
N-PVDF	6.343	880
A-PVDF	277.9	760
K-PVDF	430.5	720
S-PVDF	5.716	680

## 3. Results and Discussion

### 3.1. Optimization of the Supporting Substrate and PVDF Concentration

**Figure 2:**
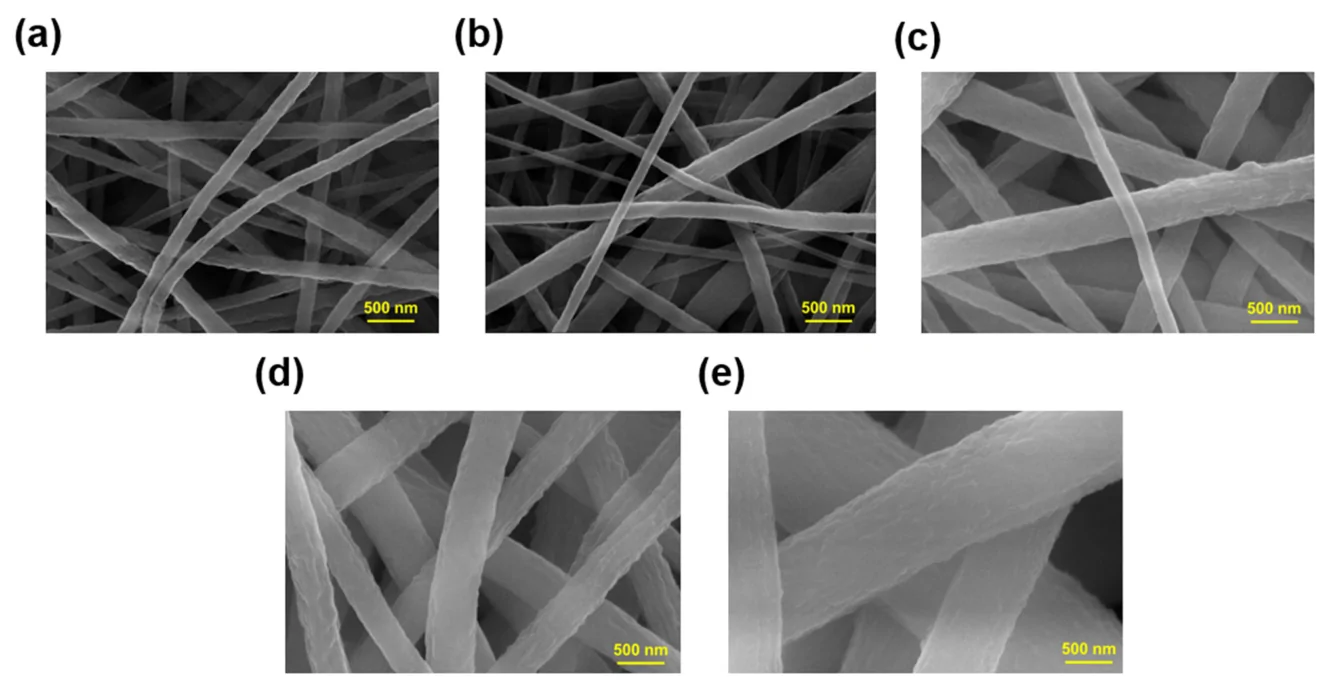
SEM images of electrospun PVDF nanofibers fabricated with various PVDF concentrations: (**a**) 7 wt.%, (**b**) 8 wt.%, (**c**) 10 wt.%, (**d**) 12 wt.%, and (**e**) 18 wt.%.

Prior to electrospinning, PP and PET non-woven substrates were evaluated as supporting layers in terms of nanofiber attachment and PP substrates exhibited superior fiber adhesion and mechanical integrity and were therefore selected as the optimal support for subsequent fabrication while PET substrates were not feasible to use as they were easily detached from the fibers.The influence of PVDF concentration on nanofiber morphology was first examined to determine an appropriate precursor composition. As shown in Figure 2, no obvious bead formation was observed over the tested concentration range; however, the nanofibers prepared at low PVDF concentrations (7–10 wt.%) showed less uniform morphology and broader diameter variation. In contrast, the 18 wt.% solution produced relatively thick fibers. Among the tested concentrations, 12 wt.% PVDF yielded the most uniform fiber morphology and was therefore selected for subsequent experiments.

### 3.2. Effects of Solution Properties and Electrospinning Conditions on Fiber Morphology

The incorporation of inorganic additives changed the conductivity and viscosity of the spinning solutions and consequently affected the applied voltage required for stable electrospinning and the resulting nanofiber structure. As summarized in Table 3, N-PVDF showed a conductivity of 6.343 μS cm^−1^ and a viscosity of 880 cP. The addition of AlCl_3_ and KNO_3_ markedly increased the conductivity to 277.9 and 430.5 μS cm^−1^, respectively, while decreasing the viscosity to 760 and 720 cP. AlCl_3_ and KNO_3_ are salt-type additives that provide mobile ionic species in the polar DMF/acetone solvent system, thereby increasing the charge-carrying ability of the spinning solutions. In contrast, Si_3_N_4_ is dispersed as solid particles rather than dissociated into mobile ions. Therefore, S-PVDF maintained a conductivity of 5.716 μS cm^−1^, similar to that of N-PVDF, while showing the lowest viscosity of 680 cP. The lower viscosities of the filler-containing solutions indicate that the fillers changed the solvation, effective chain entanglement, and flow behavior of the PVDF solutions. A similar increase in conductivity accompanied by a decrease in viscosity has been reported for PVDF solutions containing a low concentration of an ionic additive [33].

Conductivity and viscosity affect electrospinning through different mechanisms. Higher conductivity increases the charge carried by the jet and strengthens Coulombic repulsion and whipping motion, thereby promoting jet elongation and generally reducing fiber diameter. Angammana and Jayaram reported that increasing solution conductivity through NaCl addition initially increased the electrospinning jet current and resulted in a smaller average fiber diameter and improved fiber-diameter uniformity [34]. A similar fiber-thinning effect caused by salt-induced conductivity enhancement was also reported by Topuz et al. [35]. In contrast, viscosity represents the viscoelastic resistance of the solution against electrical stretching. A highly viscous solution with strong polymer-chain entanglement generally produces thicker fibers, whereas a lower viscosity facilitates jet stretching and fiber thinning. However, when the solution viscosity is too low to provide sufficient chain entanglement, the electrospinning jet becomes susceptible to capillary instability and breakup, resulting in beads, discontinuous fibers, or a broader fiber-diameter distribution [36,37]. Because the four formulations had different conductivity and viscosity values, the voltage required to establish a stable Taylor cone and continuous jet was also different. Therefore, the flow rate, nozzle size, tip-to-collector distance, and spinning time were kept constant, while the applied voltage was empirically adjusted for each formulation. N-PVDF and A-PVDF formed stable continuous jets at 21 kV. The highly conductive K-PVDF solution maintained stable fiber formation at 18 kV, whereas the low-conductivity S-PVDF solution required 21.7 kV for continuous spinning. The applied voltage was therefore adjusted to obtain a stable Taylor cone and continuous fiber-forming jet for each formulation, rather than to intentionally introduce different processing conditions. The dependence of the droplet and Taylor-cone shapes, jet mode, and resulting fiber morphology on the applied voltage has also been experimentally demonstrated by Deitzel et al. [38].

**Figure 3:**
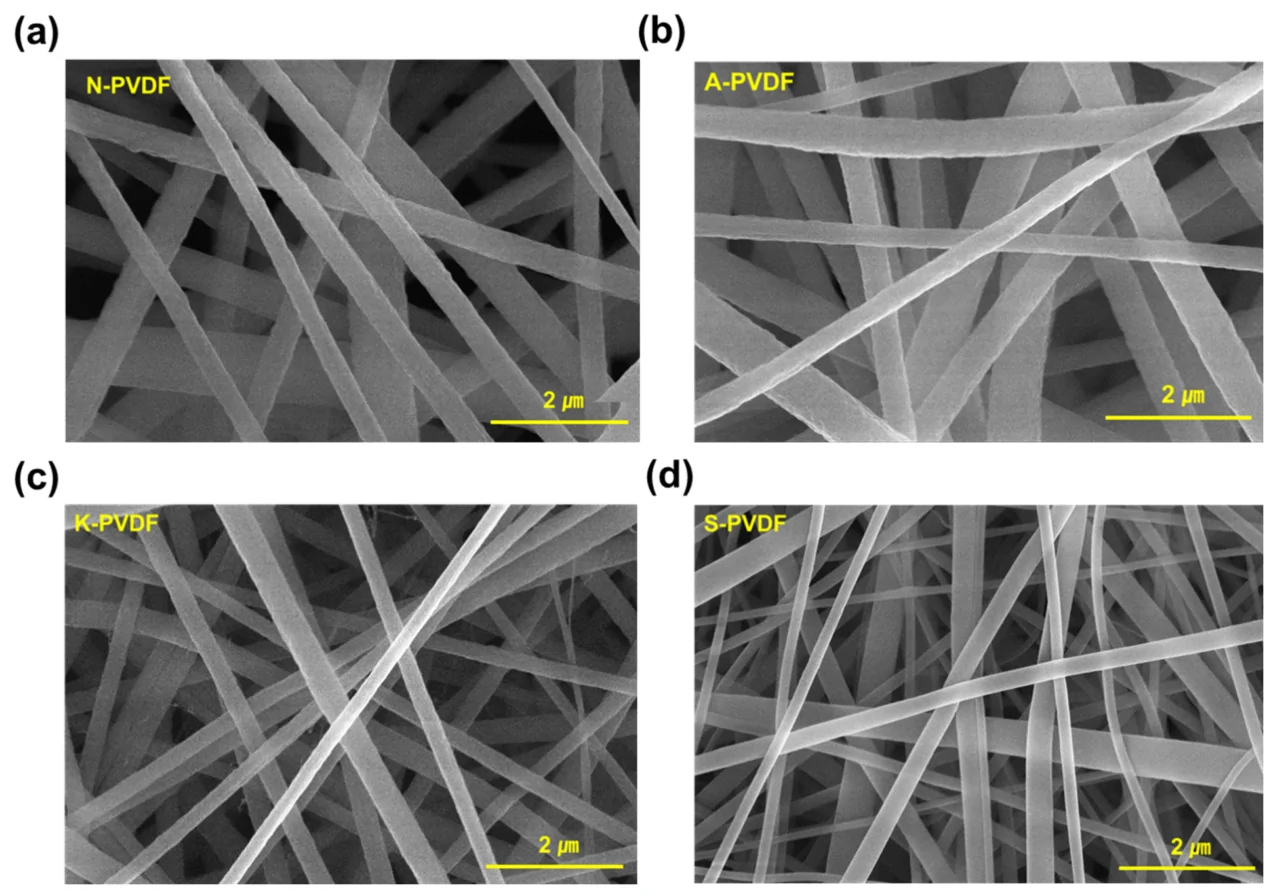
Surface SEM images of electrospun PVDF nanofibers containing different inorganic additives: (**a**) N-PVDF, (**b**) A-PVDF, (**c**) K-PVDF, and (**d**) S-PVDF. Scale bars: 2 μm.

**Figure 4:**
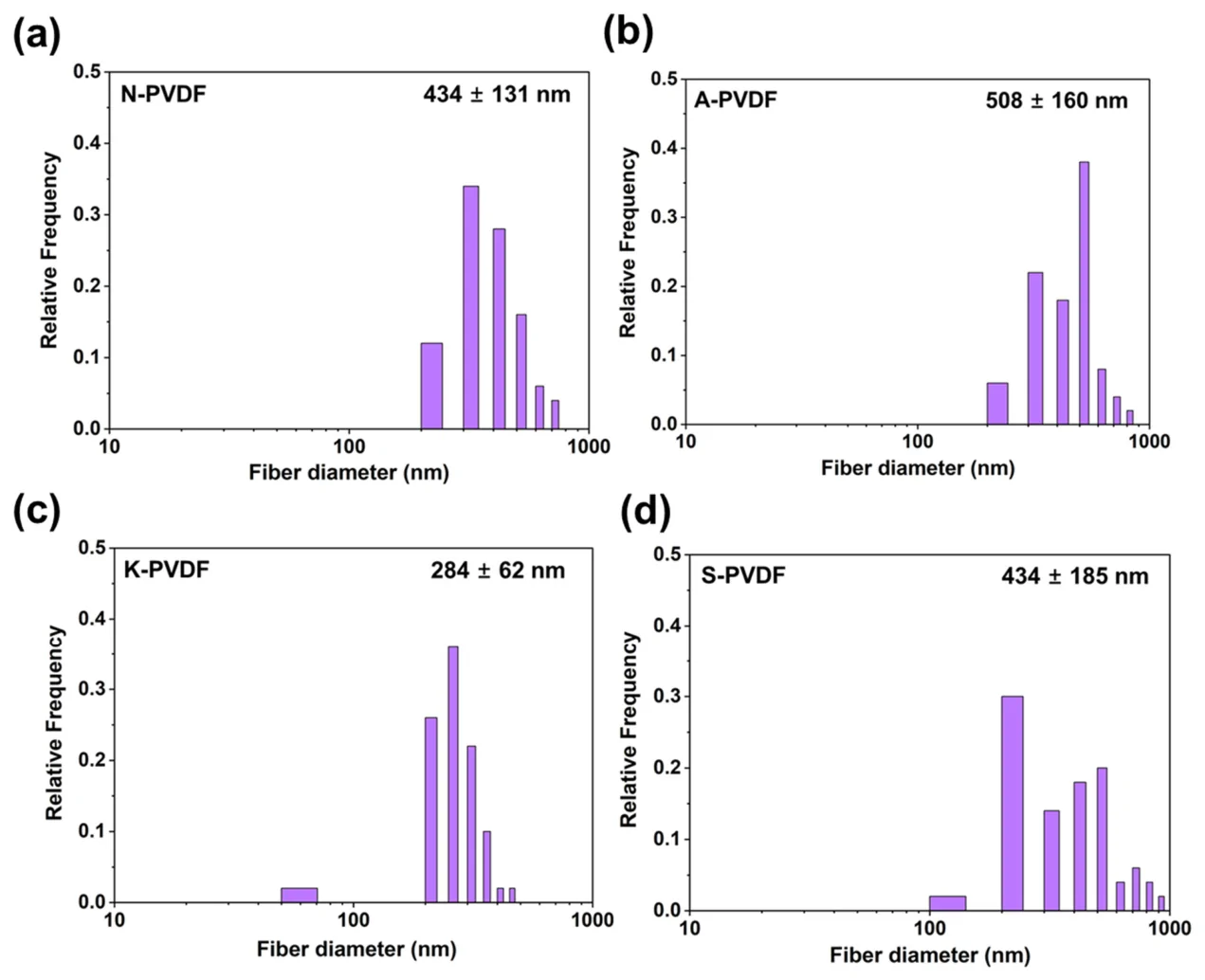
Fiber-diameter distributions of electrospun PVDF nanofiber filters analyzed using ImageJ. At least 50 individual fibers were measured for each sample: (**a**) N-PVDF, (**b**) A-PVDF, (**c**) K-PVDF, and (**d**) S-PVDF.

These differences in solution properties and stable spinning conditions were directly reflected in the fiber morphology shown in Figure 3 and Figure 4. The average fiber diameters of N-PVDF, A-PVDF, K-PVDF, and S-PVDF were 434 ± 131, 508 ± 160, 284 ± 62, and 434 ± 185 nm, respectively (Table 4). N-PVDF had the lowest conductivity and the highest viscosity, resulting in relatively limited jet stretching and the formation of comparatively thick fibers. In K-PVDF, KNO_3_ increased the conductivity to 430.5 μS cm^−1^ and decreased the viscosity to 720 cP. The increased jet charge and reduced viscoelastic resistance promoted jet elongation, producing the thinnest and most uniform fibers among the four samples. In contrast, A-PVDF showed the largest average fiber diameter and a broad diameter distribution despite its increased conductivity and reduced viscosity. Thus, the general fiber-thinning tendency expected from increased conductivity was not directly observed for A-PVDF. The introduction of Al-containing ionic species also changed the solution structure and jet stability, resulting in less uniform stretching and the formation of thicker fibers in the present PVDF system. Previous work similarly showed that AlCl_3_·6H_2_O addition changed the charge, elongation, and final morphology of electrospun P(VDF-HFP) fibers [22]. Si_3_N_4_ addition decreased the solution viscosity without increasing its conductivity. Consequently, the resistance against stretching decreased, but the electrostatic stretching force was not enhanced as it was in K-PVDF. The particle-containing jet therefore underwent less uniform stretching, producing the same average fiber diameter as N-PVDF but the broadest diameter distribution of 434 ± 185 nm.

The filler-induced changes in fiber formation and deposition behavior were further reflected in the surface pore structure. The apparent pore sizes of N-PVDF, A-PVDF, K-PVDF, and S-PVDF were 0.454 ± 0.215, 0.488 ± 0.242, 0.527 ± 0.345, and 0.332 ± 0.189 μm, respectively, while their projected pore-area fractions were 13.50, 12.80, 36.07, and 21.84%, respectively (Table 4). K-PVDF had the smallest and most uniform fibers but showed the largest apparent pore size and the highest pore-area fraction. Thus, the fine K-PVDF fibers formed an open network with relatively large spaces between the fibers rather than a densely packed surface layer. A-PVDF had the largest fiber diameter but the lowest pore-area fraction, showing that the thick fibers overlapped and occupied a large portion of the observed surface area. S-PVDF showed the smallest apparent pore size but a higher pore-area fraction than N-PVDF and A-PVDF. The coexistence of fibers with different diameters produced a network containing numerous small surface openings, resulting in the combination of a small apparent pore size and a relatively high pore-area fraction.

The cross-sectional SEM images also showed clear differences in the deposited nanofiber layers (Figure S1). The representative thicknesses of N-PVDF, A-PVDF, K-PVDF, and S-PVDF were 19 ± 4.04, 28 ± 4.24, 26 ± 6.05, and 27 ± 7.23 μm, respectively. Although the same flow rate and spinning time were used, all filler-containing filters formed thicker nanofiber layers than N-PVDF. AlCl_3_ addition produced an A-PVDF layer consisting of thick fibers and a low pore-area fraction, whereas KNO_3_ addition produced a K-PVDF layer consisting of fine fibers and a high pore-area fraction. Si_3_N_4_ addition produced relatively thick nanofiber layer with the boradest fiber-diameter distribution and numerous small surface pores, whereas A-PVDF exhibited the highest mean layer thickness. Accordingly, KNO_3_ addition resulted in a highly conductive and less viscous spinning solution and produced thin, uniform fibers with an open surface network. AlCl_3_ addition increased the solution conductivity but produced the thickest fibers and the lowest pore-area fraction in the present PVDF system. Si_3_N_4_ addition reduced the solution viscosity without increasing its conductivity and produced a thick nanofiber layer with a broad fiber-diameter distribution and small apparent surface pores.

### 3.3. Chemical Composition and Crystalline Structure of PVDF Nanofiber Filters

**Figure 5:**
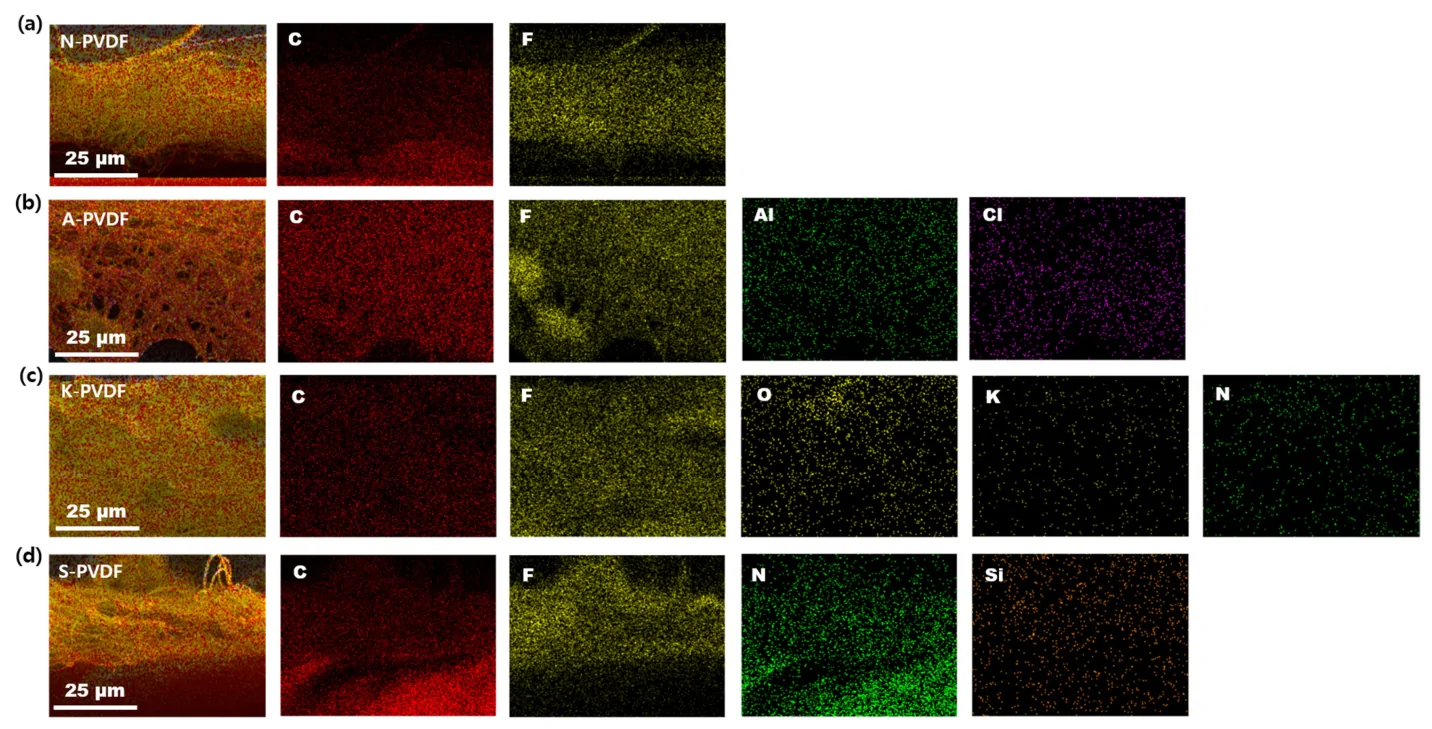
Cross-sectional SEM–EDX elemental mapping images of (**a**) N-PVDF, (**b**) A-PVDF, (**c**) K-PVDF, and (**d**) S-PVDF nanofiber filters. The corresponding elemental maps show the distributions of C and F in all filters; Al and Cl in A-PVDF; O, K, and N in K-PVDF; and N and Si in S-PVDF. Scale bars: 25 μm.

The incorporation and distribution of the inorganic additives were examined by cross-sectional SEM–EDX elemental mapping and quantitative EDX analysis. As shown in Figure 5C,F, which are the main elements of PVDF, were observed throughout all nanofiber layers. In A-PVDF, Al and Cl were distributed across the nanofiber layer, and the corresponding contents were 0.35 and 0.28 wt.%, respectively (Table 5). K-PVDF exhibited K- and O-containing regions with measured contents of 0.13 and 3.24 wt.%, respectively. An N signal was also observed in the elemental map of K-PVDF, although its content was below the quantitative reporting level in the selected EDX analysis area. In S-PVDF, both N and Si were detected, with contents of 4.66 and 0.10 wt.%, respectively. These sample-specific elemental distributions show that Al- and Cl-containing species, K-containing species, and Si- and N-containing particles remained in the corresponding PVDF nanofiber layers after electrospinning. The relatively low measured contents of Al, K, and Si are consistent with the small amounts of additives used in the spinning solutions. Differences in the measured C and F contents among the filters may also reflect the sampling of the PVDF nanofiber layer together with the underlying carbon-rich support in the cross-sectional EDX analysis.

**Figure 6:**
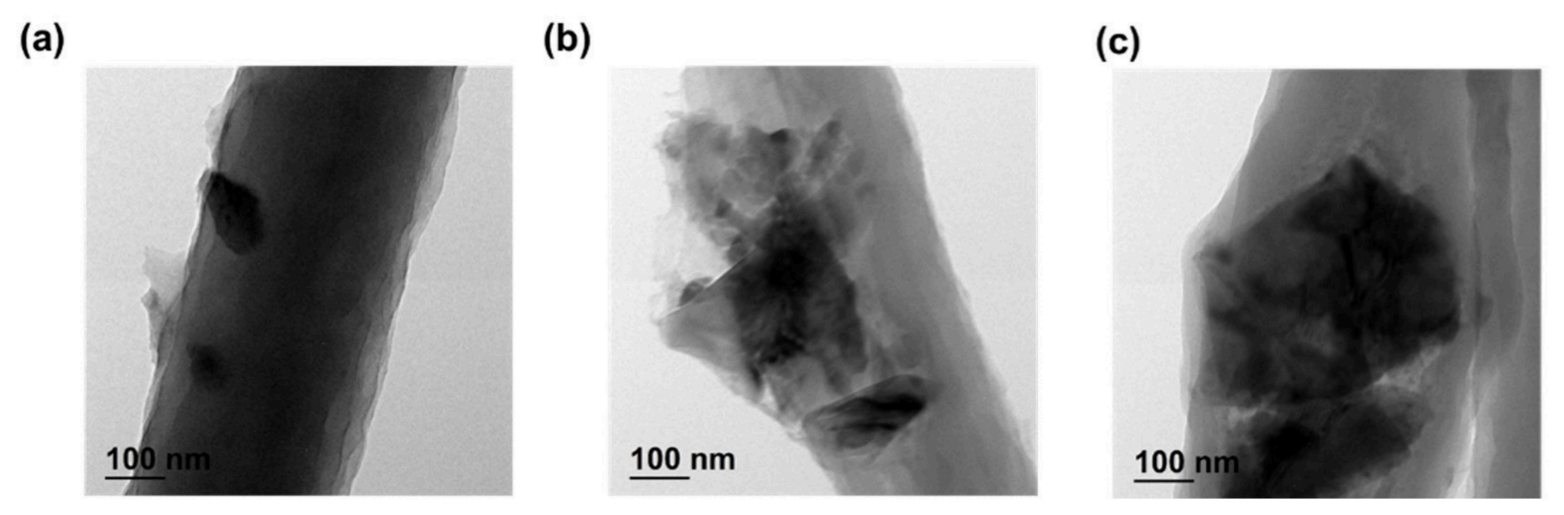
TEM images of electrospun PVDF nanofibers with different inorganic additives incorporation: (**a**) A-PVDF, (**b**) K-PVDF, and (**c**) S-PVDF nanofibers.

The TEM images further show how the inorganic additives were distributed within the individual PVDF fibers (Figure 6). Darker inorganic-rich domains were observed inside all additive-containing fibers because the inorganic components produced stronger electron contrast than the surrounding PVDF matrix. A-PVDF contained relatively small and isolated dark domains, indicating that the Al-containing species were finely distributed within the fiber. This distribution is consistent with the strong interaction of ionic Al-containing species with the polar solvent and PVDF chains in the spinning solution. In comparison, K-PVDF contained larger and more concentrated dark regions. KNO_3_ was initially introduced as a salt, but local salt-rich domains could form during rapid DMF/acetone evaporation as the solvent was removed from the electrospinning jet. S-PVDF also exhibited relatively large particulate domains embedded in the fiber, reflecting the particulate nature and local aggregation of Si_3_N_4_. Therefore, AlCl_3_ addition produced relatively small Al-rich domains, whereas KNO_3_ and Si_3_N_4_ addition produced more clearly distinguishable inorganic-rich regions within the PVDF fibers.

**Figure 7:**
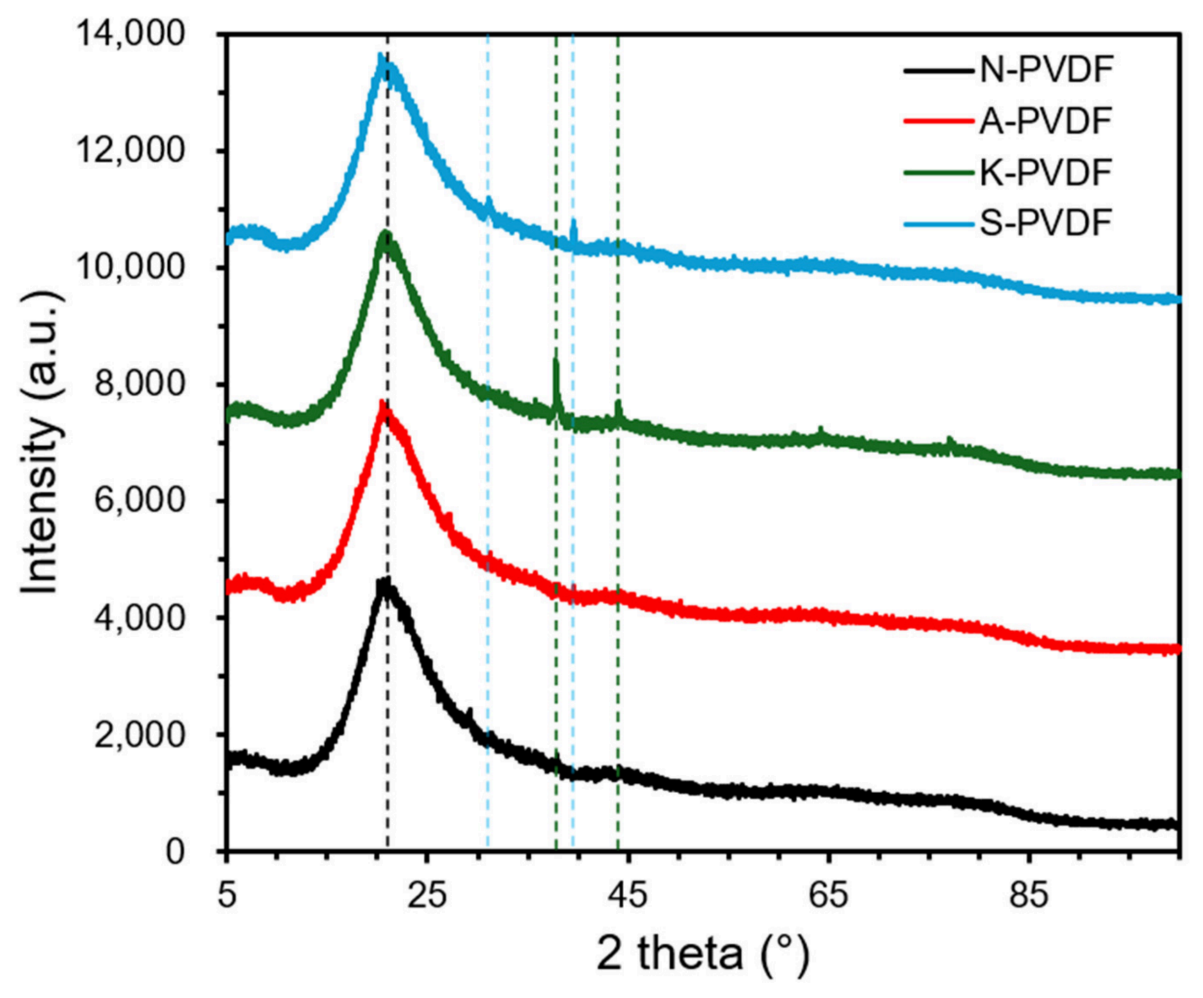
XRD patterns of N-PVDF, A-PVDF, K-PVDF, and S-PVDF nanofiber filters. All samples exhibited a broad PVDF crystalline reflection at approximately 20.4–20.7°, indicated by the black dashed line. The green and blue dashed lines indicate the characteristic diffraction positions assigned to KNO_3_ and Si_3_N_4_, respectively. The diffraction patterns were vertically offset for clarity.

XRD analysis showed that all filters exhibited a broad primary reflection at approximately 20.4–20.7° (Figure 7). This reflection is consistent with the overlapping (110)/(200) planes of β-phase PVDF near 20.6°, although a contribution from the γ-phase reflection near 20.3–20.4° cannot be excluded [39,40]. The peak positions were 20.68°, 20.66°, 20.60°, and 20.42° for N-PVDF, A-PVDF, K-PVDF, and S-PVDF, respectively. The presence of the common broad reflection in all samples shows that the principal crystalline structure of electrospun PVDF was retained after the addition of AlCl_3_, KNO_3_, and Si_3_N_4_. The absence of a large shift in this reflection also indicates that the additives were mainly distributed within the fibrous matrix rather than forming a new PVDF crystalline structure.

No distinct additional crystalline peak was observed for A-PVDF. Combined with the relatively small domains observed by TEM, this result shows that the Al- and Cl-containing species did not form a strongly diffracting crystalline phase within the PVDF fibers. In contrast, K-PVDF exhibited weak additional reflections that were consistent with the presence of crystalline KNO_3_ [41]. S-PVDF also showed weak additional features near 31.1° and 39.5°, which were located close to the diffraction regions reported for crystalline Si_3_N_4_ [42]. The additional reflections in K-PVDF and S-PVDF agree with the locally concentrated inorganic-rich domains observed by TEM. Thus, the combined SEM–EDX, TEM, and XRD results show that the three additives were incorporated in different forms: AlCl_3_ produced finely distributed Al- and Cl-containing species without distinct crystalline reflections, KNO_3_ remained as locally concentrated crystalline salt domains, and Si_3_N_4_ remained as ceramic particulate domains embedded within the PVDF fibers.

### 3.4. Surface Characterization of PVDF-Based Electrospun Nanofiber Filters

**Figure 8:**
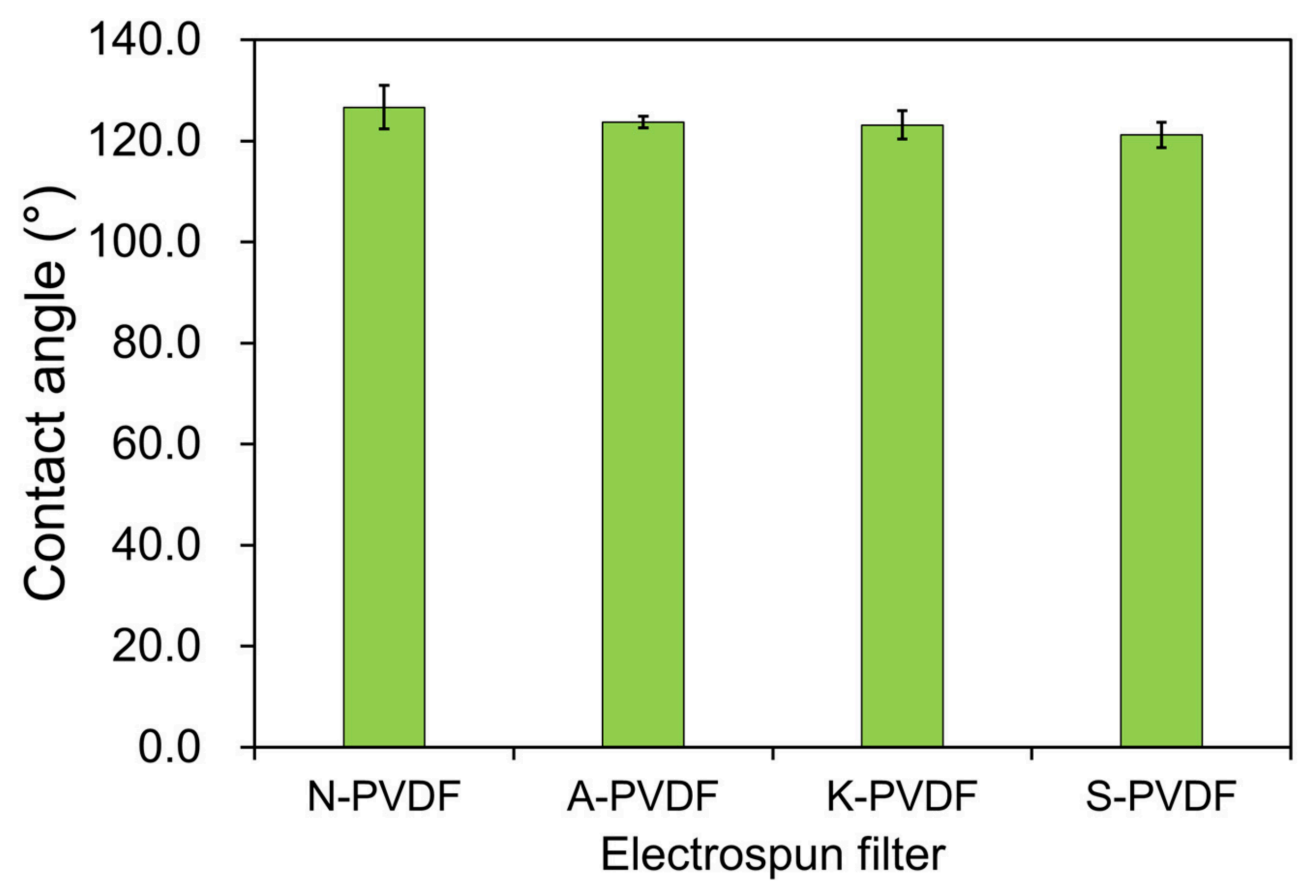
Surface wettability of electrospun PVDF nanofibers.

In addition to morphology, water contact angle was measured to evaluate the surface wettability of the electrospun filters. This analysis was included because surface wettability can affect the interaction of fibrous filters with moisture, condensed droplets, and humid airborne particulates under practical operating conditions [43]. In PM filtration systems, hydrophobic surfaces are often considered beneficial for suppressing water accumulation within the fibrous network and for maintaining filtration stability under humid conditions. Previous PM-filter studies have likewise used contact angle measurements to assess the moisture resistance of nanofibrous air filters, particularly when hydrophobic or superhydrophobic layers were introduced to improve durability in moisture-containing environments [44]. As shown in Figure 8, only slight differences in contact angle were observed after additive incorporation, and all samples exhibited contact angles above 120°. These results indicate that the hydrophobic character of PVDF was largely preserved regardless of additive type. Therefore, although contact angle measurement is useful to confirm the moisture-resistant surface nature of the filters, the small variation among the samples suggests that surface wettability was not the primary factor governing the differences observed in their later electrostatic behavior.

**Figure 9:**
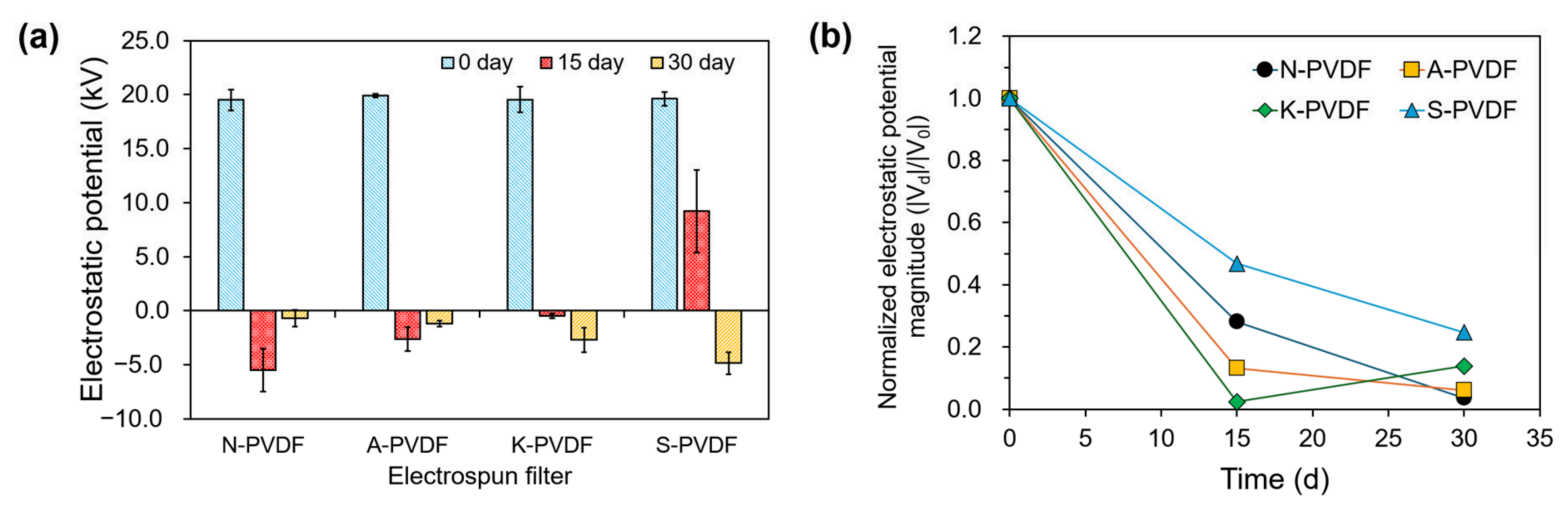
Time-dependent evolution of electrostatic potential and charge retention in neat and additive-modified electrospun PVDF nanofiber filters. (**a**) Measured electrostatic potentials at different aging times. (**b**) Normalized electrostatic potential magnitude (|V_d_|/|V_0_|) illustrating differences in charge decay behavior among the filters.

The electrostatic behavior of the filters was further examined by monitoring the surface potential during aging (Figure 9), since electrostatic adsorption is a key contributor to the capture of fine particulate matter in electret-type filters. Prior work on PVDF-based air filters has shown that filtration efficiency is closely related to surface electrostatic potential, and that the decay of stored charges over time or under humid conditions directly reduces electrostatic adsorption capability [45]. For this reason, surface potential measurement is widely used as a meaningful indicator of electret performance and long-term filtration stability [46]. As shown in Figure 9a, all samples exhibited similar initial electrostatic potentials at 0 day, indicating that the initial charge generation during electrospinning was broadly comparable. However, clear differences emerged during aging. N-PVDF, A-PVDF, and K-PVDF showed substantial charge decay with time, whereas S-PVDF retained a considerably higher residual potential after both 15 and 30 days. The normalized data in Figure 9b further confirm that S-PVDF showed the slowest charge decay, while K-PVDF decayed most rapidly; electrostatic filtration is governed more directly by the magnitude of the stored electrostatic field than by charge polarity alone, the absolute value of the surface potential was considered here to evaluate charge-retention behavior among the filters [47]. Since the contact angles of all filters were similar, these differences may be attributed to differences in charge trapping and dissipation behavior within the fiber matrix than to differences in surface wettability. This interpretation is consistent with previous electret-filter studies in which filler composition and dielectric characteristics strongly influenced retained surface potential and filtration durability [24].

For the present samples, the superior charge retention of S-PVDF suggests that the incorporated Si-containing additive helped stabilize the stored electrostatic charges, whereas K-PVDF, despite showing favorable fiber diameter characteristics, exhibited relatively poor long-term charge preservation. Thus, Figure 8 and Figure 9 together show that the selected additive incorporation in this study had only a limited effect on hydrophobicity, but a pronounced effect on electrostatic stability, which is expected to be more relevant to long-term PM filtration performance.

### 3.5. Initial Filtration Performance and Pressure Drop Characteristics

**Figure 10:**
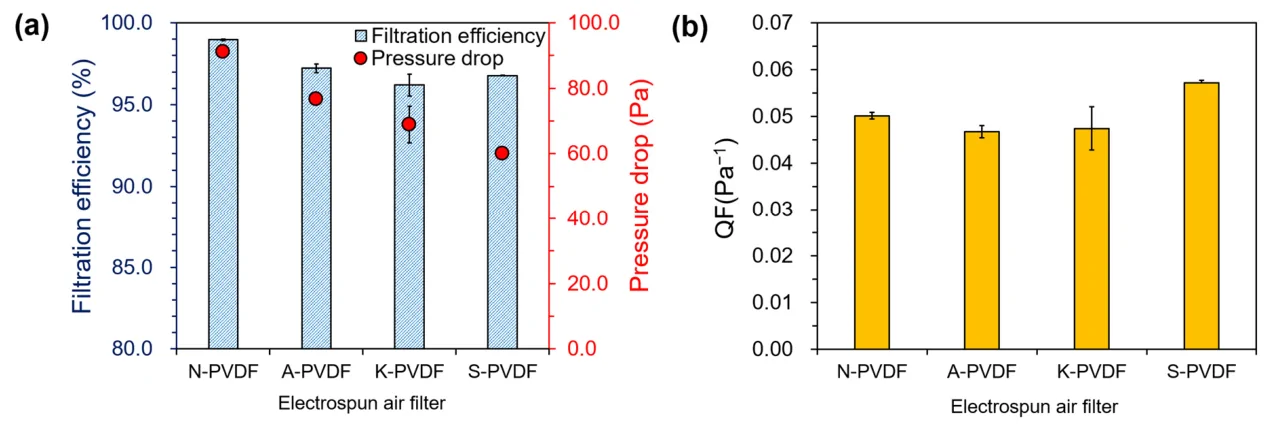
Initial performance of PVDF-based electrospun air filters. (**a**) Filtration efficiency (blue, left y-axis) and pressure drop (red, right y-axis) and (**b**) QF.

The initial filtration efficiencies of N-PVDF, A-PVDF, K-PVDF, and S-PVDF were 98.96 ± 0.04%, 97.23 ± 0.26%, 96.20 ± 0.67%, and 96.77 ± 0.02%, respectively (Figure 10a). In contrast, the pressure drops differed more clearly among the samples, with N-PVDF showing the highest pressure drop of 91.14 ± 0.98 Pa, followed by A-PVDF (76.77 ± 0.57 Pa), K-PVDF (68.93 ± 5.57 Pa), and S-PVDF (60.11 ± 0.57 Pa). The contribution of the PP supporting layer to the measured pressure drop was considered negligible. In our previous study, the same type of bare PP supporting fabric exhibited a filtration efficiency of only 8.23% with a negligible pressure drop of approximately 0 Pa when evaluated using a TSI 8130 automated filter tester at a face velocity of 5.3 cm s^−1^ over a test area of 100 cm^2^ [4]. These conditions were essentially the same as those used in the present study. Therefore, the differences in filtration efficiency and pressure drop among the present filters were predominantly associated with the deposited PVDF nanofiber layers rather than with the PP supporting layer. Accordingly, S-PVDF exhibited the highest QF, whereas N-PVDF showed the lowest QF among the tested filters (Figure 10b). These results indicate that, although all samples provided similarly high initial particle removal efficiency, their overall filtration performance differed in terms of airflow resistance. Such differences are likely associated with additive-induced changes in fiber morphology and packing structure, as observed in Figure 3 and Figure 4. According to previous studies on electrospun air filters, pressure drop is strongly influenced by structural parameters such as fiber diameter, filter thickness, porosity, and packing density, because these factors determine the available airflow pathways through the fibrous network [48,49]; The relatively low pressure drop of S-PVDF may be associated with the combined effects of its fiber-diameter distribution, apparent surface pore structure, and nanofiber-layer thickness. Because the deposited mass per unit area, bulk porosity, and packing density were not quantitatively determined, their individual contributions to the measured pressure drop could not be separately evaluated.

In addition, the superior QF of S-PVDF appears to result from the combination of its low airflow resistance and the preservation of sufficiently high initial filtration efficiency. In this regard, the role of the Si_3_N_4_ additive may extend beyond structural modification. Previous electret-filter studies have reported that Si_3_N_4_ can act as an electret-promoting component in fibrous air filters by improving charge-storage behavior and electrostatic adsorption [24,25]. For example, PU/Si_3_N_4_ electret nanofiber membranes have been reported to maintain favorable air permeability together with effective particle filtration, and moisture-/oil-stable electret filters incorporating Si_3_N_4_ have shown enhanced charge-storage capability [25].

### 3.6. Time-Dependent Filtration Stability

**Figure 11:**
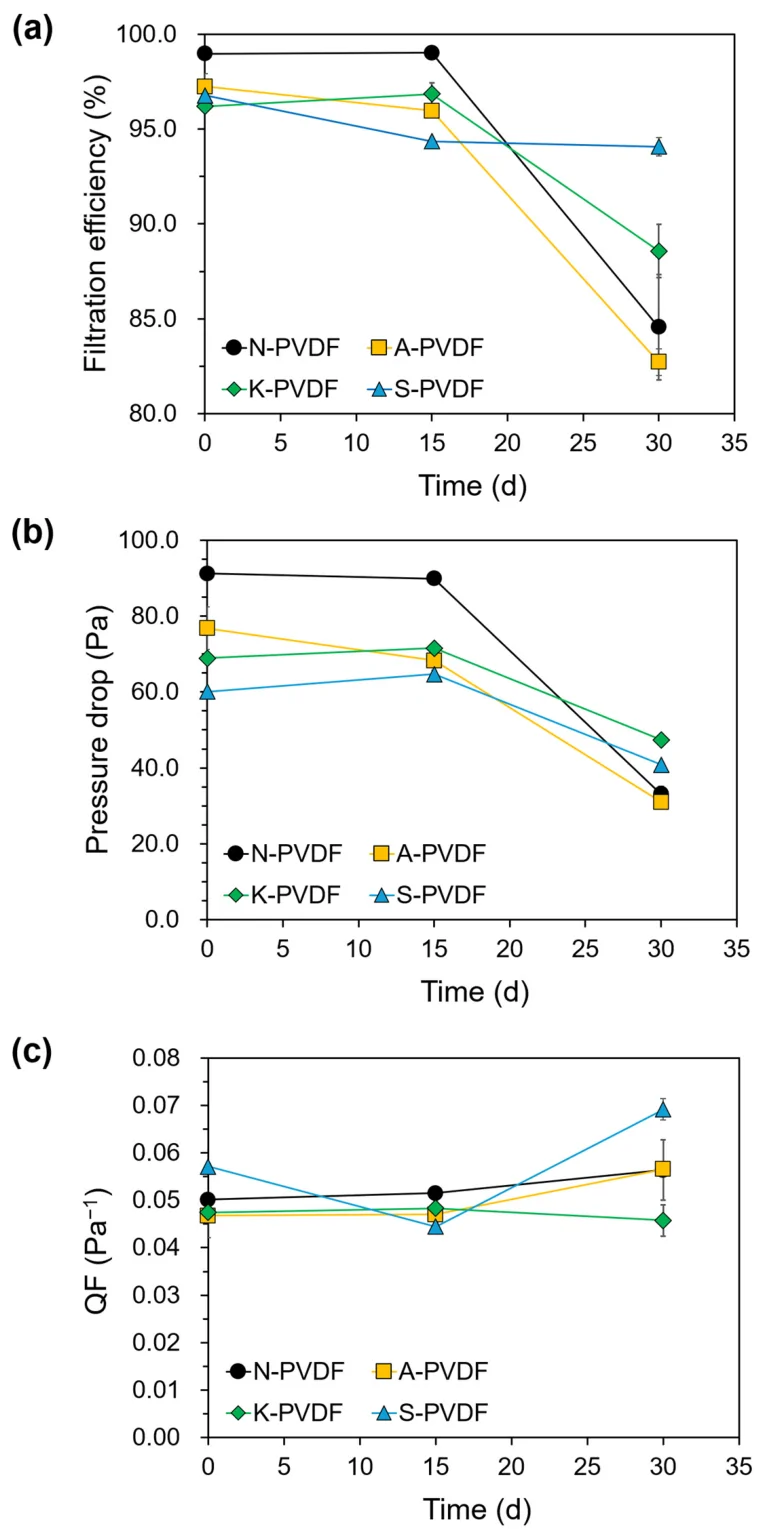
Evolution of (**a**) filtration efficiency, (**b**) pressure drop, and (**c**) QF of PVDF-based electrospun filters over time.

The time-dependent filtration stability of the electrospun PVDF-based nanofiber air filters was evaluated by monitoring changes in filtration efficiency, pressure drop, and QF after storage for 15 and 30 days, as shown in Figure 11. Although all filters exhibited some degree of performance change during storage, the extent of the change strongly depended on filter composition. After 15 days of storage, all samples still maintained relatively high filtration efficiencies, with values of 99.02 ± 0.12% for N-PVDF, 95.96 ± 0.58% for A-PVDF, 96.84 ± 0.04% for K-PVDF, and 94.35 ± 0.13% for S-PVDF (Figure 11a). Their corresponding pressure drops were 89.83 ± 0.57 Pa, 68.27 ± 1.13 Pa, 71.54 ± 0.98 Pa, and 64.68 ± 0.98 Pa, respectively (Figure 11b). At this stage, N-PVDF still exhibited the highest pressure drop, whereas S-PVDF maintained the lowest pressure drop among the tested filters.

More distinct changes were observed after 30 days. The filtration efficiencies of N-PVDF, A-PVDF, K-PVDF, and S-PVDF decreased to 84.57 ± 2.77%, 82.73 ± 1.40%, 88.57 ± 0.70%, and 94.07 ± 0.49%, respectively, while the corresponding pressure drops decreased to 33.15 ± 1.98, 31.05 ± 1.13, 47.40 ± 0.57, and 40.86 ± 0.57 Pa (Figure 11a,b). N-PVDF and A-PVDF exhibited pressure-drop reductions of approximately 64% and 60%, respectively, accompanied by filtration-efficiency losses of 14.39 and 14.50 percentage points. K-PVDF showed intermediate decreases, whereas S-PVDF exhibited a pressure-drop reduction of approximately 32% but maintained a filtration efficiency of 94.07%, corresponding to a decrease of only 2.70 percentage points. The simultaneous decreases in pressure drop and filtration efficiency are most consistent with a reduction in the effective airflow resistance of the deposited nanofiber layer. During storage, relaxation or local rearrangement of the loosely deposited electrospun fibers may have produced more open or preferential airflow pathways through the nanofiber layer. Such a change would allow air to pass through the filter with lower resistance while reducing the probability of particle–fiber contact, thereby decreasing both pressure drop and mechanical particle capture.

Previous studies have shown that changes in the thickness, pore structure, and physical integrity of fibrous filter layers can alter their airflow resistance and particle-capture performance. Lee et al. observed lower pressure drops after extended thermal or humid storage of PP electret filters. Their structural analysis showed enlarged pores after thermal treatment, while fiber breakage was observed under severe recurrent storage and particle-loading conditions [50]. Xu et al. also reported that fiber vibration and the resulting expansion of the fibrous pore structure reduced the pressure drop of an electrospun PVDF-based filter from 96 to 45 Pa [51]. Although the storage and treatment conditions used in these studies differed from those employed in the present work, their results demonstrate that changes in the physical structure and airflow pathways of a fibrous filter can produce a substantial reduction in pressure drop. In the present study, the decrease occurred in all four filters but differed in magnitude depending on the filter composition and initial fibrous structure. The concurrent decreases in pressure drop and filtration efficiency are therefore consistent with a change in the effective airflow pathways of the deposited nanofiber layers during storage. Charge decay alone cannot explain the marked reduction in pressure drop because charge loss primarily affects electrostatic particle capture and causes only a minor change in airflow resistance when the fibrous web structure remains intact [52].

The differences among the filters further show that the reduction in physical airflow resistance alone did not determine filtration-efficiency retention. Although S-PVDF showed a pressure-drop reduction similar to that of K-PVDF, it maintained a substantially higher filtration efficiency and the highest normalized surface potential after 30 days, as shown in Figure 9. Previous studies on PVB/Si_3_N_4_- and PU/Si_3_N_4_-based electret filters have shown that Si_3_N_4_ can contribute to surface-potential retention and electrostatic particle capture [24,25] The higher retained surface potential of S-PVDF therefore helped maintain its electrostatic particle-capture contribution despite the decrease in physical airflow resistance. Thus, the filtration behavior after 30 days was associated with both the change in physical airflow resistance and the differences in electrostatic charge retention rather than with charge decay alone. The corresponding QF values calculated from the measured filtration efficiency and pressure drop are presented in Figure 11c. S-PVDF exhibited the highest QF after 30 days because it maintained a relatively high filtration efficiency while showing a reduced pressure drop. However, because the Day 30 QF values were strongly affected by the substantial reduction in pressure drop, they were not used as direct evidence of electrostatic charge stabilization.

## 4. Conclusions

In this study, the effects of different inorganic additives on electrospun PVDF-based nanofiber air filters were examined. Although all filters exhibited high initial filtration efficiencies above 96%, the additives had greater effects on pressure drop, QF, and long-term filtration stability than on initial filtration efficiency. These differences were associated with additive-induced changes in fiber structure and electrostatic charge-retention behavior during storage. Among the tested samples, S-PVDF retained the highest filtration efficiency and normalized surface-potential magnitude after 30 days. S-PVDF also exhibited the highest Day 30 QF, although this value was affected by the substantial decrease in pressure drop. Overall, the improved filtration-efficiency retention of S-PVDF was associated with its more stable electrostatic charge-retention behavior.

## Figures and Tables

**Table 4:** Structural characteristics of neat and inorganic additive-incorporated PVDF nanofiber filters estimated from surface, cross-sectional SEM images and a high precision digital micrometer.

Sample	Average Fiber Diameter (nm)	Apparent Pore Size (μm)	Projected Pore-Area Fraction (%)	Representative Thickness (μm)
N-PVDF	434 ± 131	0.454 ± 0.215	13.50	19 ± 4.04
A-PVDF	508 ± 160	0.488 ± 0.242	12.80	28± 4.24
K-PVDF	284 ± 62	0.527 ± 0.345	36.07	26 ± 6.05
S-PVDF	434 ± 185	0.332 ± 0.189	21.84	27 ± 7.23

Note: Apparent pore size and projected pore-area fraction were estimated from two-dimensional top-view SEM images using ImageJ. Thickness values represent nanofiber-layer thicknesses measured using a high-precision digital micrometer.

**Table 5:** EDX elemental composition (wt.%) of neat and inorganic filler incorporated PVDF air filters.

Element	N-PVDF	A-PVDF	K-PVDF	S-PVDF
C	69.29	66.34	55.24	68.08
F	30.71	33.03	41.39	27.16
Al	-	0.35	-	-
Cl	-	0.28	-	-
K	-	-	0.13	-
N	-	-	-	4.66
O	-	-	3.24	-
Si	-	-	-	0.10
Total	100.0	100.0	100.0	100.0

## Data Availability

The original contributions presented in this study are included in the article/Supplementary Material. Further inquiries can be directed to the corresponding authors.

## Supplementary Materials

The following supporting information can be downloaded at: https://www.mdpi.com/article/10.3390/nano16161023/s1. Figure S1. Cross-sectional SEM images of electrospun PVDF nanofiber filters: (a) N-PVDF, (b) A-PVDF, (c) K-PVDF, and (d) S-PVDF. Scale bar: 100 μm.
