# Supplementary material for: Comparative Effects of Inorganic Additives on the Filtration Performance of PVDF-Based Electrospun Air Filters
Source: Nanomaterials (Basel). 2026 Aug 18;16(16):1023. doi: 10.3390/nano16161023 (PMC13516307; doi:10.3390/nano16161023)
Supplement: Supplementary file 1 [file nanomaterials-16-01023-s001.zip › nanomaterials-4425951-supplementary.pdf]

Supplementary File

Comparative effects of inorganic additives on the filtration performance of PVDF-based electrospun air filters

Chanwoo Park [1], Dohyoung Kang [1], Hobin Jee [1], Yebin Hong [1], Changhyuk Kim [2,3], Sukbyung Chae [4], Jungmin Lee [5], Soonchul Kwon [2], Ji Yong Park [6], Numan Yanar [7,*] and Euntae Yang [1,*]

[1]Department of Marine Environmental Engineering, Gyeongsang National University, Tongyoung 53064, Republic of Korea; pcw0112@gnu.ac.kr (C.P.); ybh03@gnu.ac.kr (Y.H.)

[2]Department of Civil and Environmental Engineering, Pusan National University, Busan 46241, Republic of Korea; changhyuk.kim@pusan.ac.kr (C.K.); sckwon@pusan.ac.kr (S.K.)

[3]Institute for Environment and Energy, Pusan National University, Busan 46241, Republic of Korea

[4]Department of Mechanical Engineering, Korea University of Technology and Education, Cheonan 31253, Republic of Korea; schae@koreatech.ac.kr

[5]Land & Housing Institute, Korea Land & Housing Corporation, Daejeon 305713, Republic of Korea

[6]Nexmore Systems Co., Ltd., Ansan 15426, Republic of Korea

[7]R&D Center, Naieel Technology, Daejeon 34026, Republic of Korea

*Correspondence: numanyanar@gm.gist.ac.kr (N.Y.); yet83@gnu.ac.kr (E.Y.)

***Co-corresponding authors**

E-mail addresses: yet83@gnu.ac.kr (Prof. Euntae Yang), numanyanar@gm.gist.ac.kr (Dr. Numan Yanar)

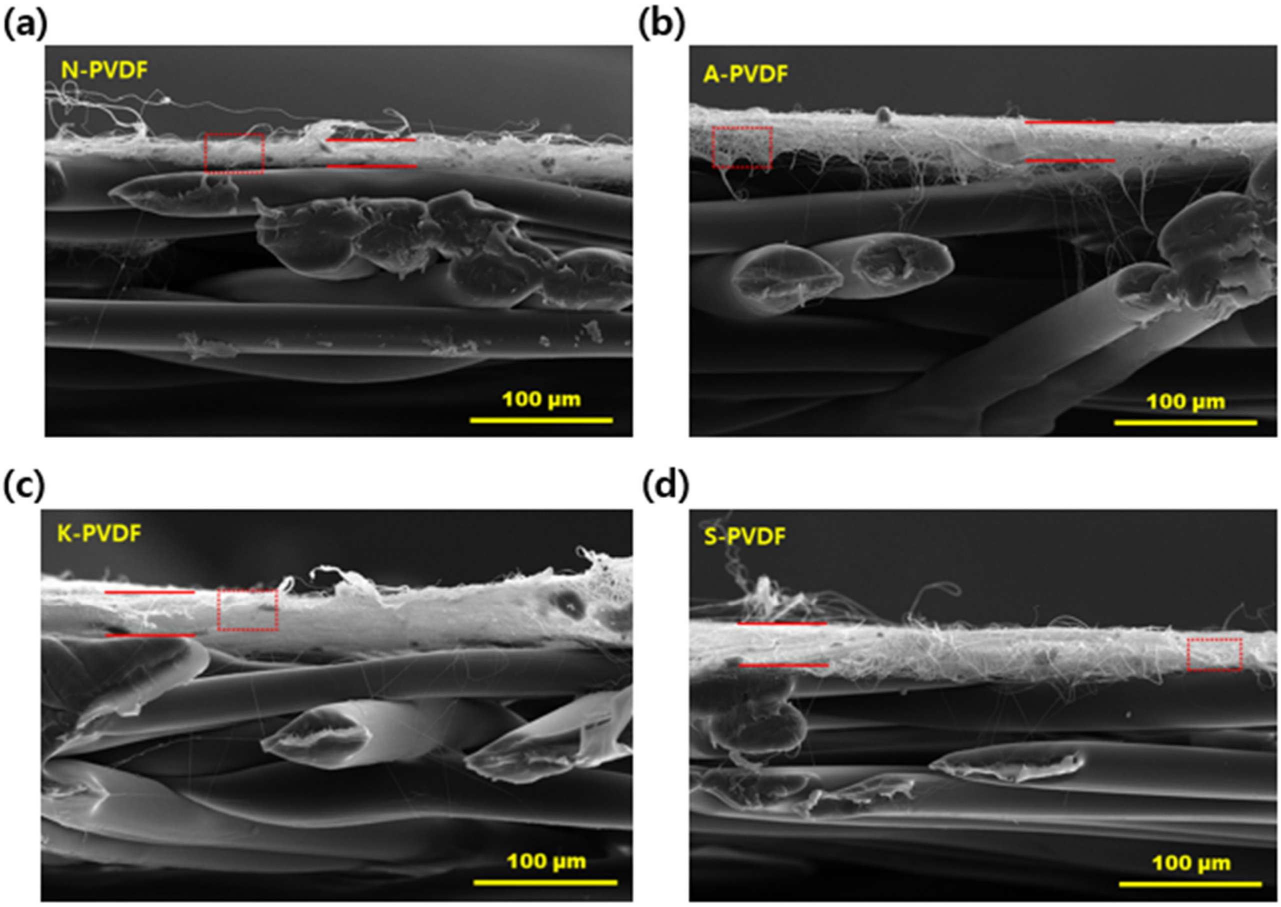

Figure S1. Cross-sectional SEM images of electrospun PVDF nanofiber filters containing different inorganic additives: (a, b) N-PVDF, (b) A-PVDF, (c) K-PVDF, and (d) S-PVDF.
